# Socioeconomic inequality in early sexual initiation among female youths in sub-Saharan African countries: a decomposition analysis

**DOI:** 10.1186/s12889-023-16124-6

**Published:** 2023-07-04

**Authors:** Elsa Awoke Fentie, Atitegeb Abera Kidie, Samrawit Mihret Fetene, Ever Siyoum Shewarega

**Affiliations:** 1 Department of Reproductive Health, Institute of Public Health, College of Medicine and Health Sciences, University of Gondar, Gondar, Ethiopia; 2 School of public health, college of health science, Woldia University, Woldia, Ethiopia; 3 Department of Health Systems and Policy, Institute of Public Health, College of Medicine and Health Sciences, University of Gondar, Gondar, Ethiopia; 4 Department of Reproductive Health, School of Public Health, College of Medicine and Health Science, Dilla University, Dilla, Ethiopia

**Keywords:** Early sexual initiation, Socioeconomic inequality, Youth, Decomposition analysis, DHS, Sub-Saharan Africa

## Abstract

**Introduction:**

Youths are defined as individuals within the age group 15–24 years. It is the transitional stage from childhood to adulthood with biological, social, and psychological change, so it is a time of risk and opportunity for their future life. Early sexual initiation exposes young people to various social, economic, sexual, and reproductive health issues, such as unwanted adolescent pregnancies, sexually transmitted infections, unsafe abortion, cervical cancer, and early marriages. Therefore, this study aimed to assess the presence of socioeconomic inequality in early sexual initiation and contributing factors in sub-Saharan African countries.

**Methods:**

A total of 118,932 weighted female youths from SSA countries’ DHS data were included in the study. Socioeconomic inequality of Early sexual initiation was evaluated using the Erreygers znormalized concentration index and associated concentration curve. Decomposition analysis was performed to determine those factors causing socioeconomic-related inequality.

**Results:**

The weighted Erreygers normalized concentration index of wealth-related inequality of early sexual initiation was − 0.157 with a Standard error = 0.0046 (*P* value < 0.0001); this indicated that early sexual initiation was disproportionately concentrated among the poor (pro-poor). Moreover, the weighted Erreygers normalized concentration index (ECI) of educational status-related inequality of early sexual initiation was − 0.205 with a Standard error = 0.0043 (*P* value < 0.0001). This indicated that early sexual initiation was disproportionately concentrated among youths with no formal education. The decomposition analysis revealed that mass media exposure, wealth index, place of residency, religion, marital status, educational status, and age were significant contributors to the pro-poor socioeconomic inequalities in early sexual initiation.

**Conclusion and recommendation:**

This study has revealed pro-poor inequality in early sexual initiation. Therefore, priority must be given to modifiable factors such as promoting the accessibility of media exposure in the household, improving the educational opportunity of female youths, and improving their country’s economy to a higher economic level to improve the wealth status of the population.

## Introduction

The World Health Organization (WHO) defines youths as individuals aged 15–24 years [1]. It is the transitional stage from childhood to adulthood with biological, social, and psychological change, so it is a time of risk and opportunity for their future life [2]. Early sexual initiation is defined as sexual activity that begins earlier than 18 years of age (3), and most often, it is considered a risky sexual behavior because of its adverse consequences [3].

Early sexual initiation exposes young people to various social, economic, sexual, and reproductive health issues, such as unwanted adolescent pregnancies, sexually transmitted infections, unsafe abortion, cervical cancer, and early marriages [4–6]. Due to their physiological immaturity in having sex and giving birth, female youths are particularly more susceptible to pregnancy and birthing complications, including fistula or even death [7, 8]. It also increases the risk of school dropout, poor school performance, stigma, and discrimination [9].

Different scholars revealed that Early sexual initiation among youths is associated with; sex, age, residence, religion, educational status, viewing pornographic material, alcohol drinking, khat chewing, media exposure, income, employment status, comprehensive HIV knowledge, knowledge of family planning, peer pressure [10–17].

Although policies, laws, and strategies are well-defined and insightful about youth’s health and both governmental and non-governmental organizations, work to ensure universal access to sexual and reproductive healthcare services for all, including family planning, information,education, and the integration of reproductive health into national strategies and programs by 2030. Youths are still experiencing early sexual initiation, unwanted pregnancy, abortion, and other sexual problems, especially in sub-Saharan African countries, and there is limited information about socioeconomic-related inequality of early sexual initiation in sub-Saharan Africa.

Therefore, this study aimed to assess the pooled prevalence of early sexual initiation, the level of socioeconomic inequalities of early sexual initiation, and contributing factors for the socioeconomic inequalities among female youths in sub-Saharan Africa (SSA) countries. This will help countries to ensure their disadvantaged populations are not left behind and help policymakers to narrow the disparity of youth sexual health by wealth status.

## Method

### Study design, setting and period

This study was conducted among Sub-Saharan Africa (SSA) countries. The sub-Saharan region is the area on the African continent that lies south of the Sahara and consists of four vast and distinct regions i.e., Eastern Africa (Burundi, Comoros, Ethiopia, Kenya, Malawi, Mozambique, Rwanda, Tanzania, Uganda, Zambia, Zimbabwe), Central Africa (Angola, Cameroon, Chad, the Democratic Republic of the Congo, Republic of the Congo, Gabon), Western Africa (Benin, Burkina Faso, Ivory Coast, Gambia, Ghana, Guinea, Liberia, Mali, Niger, Nigeria, Senegal, Sierra Leone, Togo), and Southern Africa (Lesotho, Namibia, South Africa) [18]. A recent standard DHS data set of SSA countries within 10 years (2010–2020) was our data source, a cross-sectional study conducted every 5-year interval to generate updated health and health-related indicators.

### Population

#### Source population

The source population was all-female youth (15–24 years old), irrespective of their sexual activity across SSA countries.

#### Study population

The study populations were all-female youth (15–24 years old), irrespective of their sexual activity in the selected Enumeration Areas (EA).

### Inclusion criteria

This study included all female youth (15– 24 years old) in the selected EAs in each SSA country.

### Exclusion criteria

A total of 47 countries are located in SSA. Of these countries, only 41 had DHS reports. However, the DHS data of the Central Africa Republic, Eswatini, Sao Tome Principe, Madagascar, and Sudan were conducted before 2010. Therefore we excluded those countries from further analysis. Moreover, three countries (Botswana, Mauritania, and Eritrea) were excluded because the DHS data set was not publicly available. Finally, youths’ lives in a total of 33 sub-Saharan African countries were included in this study.

### Sample size determination and sampling method

For this study, we used the most recent DHS data that was conducted from 2010 to 2020. There were 33 countries with DHS conducted in the study period. The DHS sample was stratified and selected in two stages. Every geographical region in the countries was divided into urban and rural areas to carry out stratification. In the first sampling stage, EAs were selected with probability proportional to size within each stratum, and in the second stage, following the listing of the households in the selected EAs, a fixed number of households is selected by equal probability systematic sampling [19]. Throughout the analysis, we applied weighting to restore the representativeness and to get a better statistical estimate [20].

Finally, total weighted samples of 118,932 Female youths were included from 33 selected SSA countries (Table 1).

**Table 1:** Sample size determination of early sexual initiation and factor associated with it among female youths in each sub-Saharan Africa: based on 2010–2020 DHS

Region	Country	DHS year	Weighted sample
East Africa	Burundi	2016/17	2,652
	Comoros	2012	871
	Ethiopia	2016	2,865
	Kenya	2014	7,289
	Malawi	2015/16	7,597
	Mozambique	2011	4,460
	Rwanda	2014/15	2,090
	Tanzania	2015/16	3,788
	Uganda	2016	5,470
	Zambia	2018	4,005
	Zimbabwe	2015	2,154
Central Africa	Angola	2015/16	5,041
	Cameroon	2018	3,671
	Chad	2014/15	4,712
	DR Congo	2013/14	5,496
	Congo	2011/12	3,398
	Gabon	2012	2,731
Western Africa	Benin	2017/18	4,338
	Burkina Faso	2010	4,544
	Ivory Coast	2011/12	3,195
	Gambia	2013	1,806
	Ghana	2014	2,099
	Guinea	2018	2,668
	Liberia	2019/20	2,616
	Mali	2018	2,981
	Niger	2012	3,019
	Nigeria	2018	8,852
	Senegal	2019	1,485
	Sierra Leone	2019	4,465
	Togo	2013/14	2,294
South Africa	Lesotho	2014	1,896
	Namibia	2013	2,481
	South Africa	2016	1,903

### Study variables

#### Dependent variables

Socioeconomic-related inequality in early sexual initiation was the outcome variable. In this study, early sexual initiation was dichotomized as (Yes/No). Youth who started sexual activity before 18 are considered to have early sexual initiation [21]. The covariance between early sexual initiation and the measurement of living standards distribution (wealth index) can be used to illustrate the socioeconomic inequality in early sexual initiation. Then, it was classified into either pro-poor, pro-rich, or no inequality.

#### Independent variable

In this study age of the respondent, religion, khat chewing, wealth index, educational status, marital status, employment status, mass media exposure, the region in SSA, residence, country income level, knowledge about any family planning method, ever heard about STI, and experienced sexual violence were incorporated as explanatory variables. The socioeconomic status was measured using the wealth index from DHS data sets. In the DHS data, the wealth index was constructed using principal component analysis and then categorized as poorest (quintile 1), poorer (quintile 2), middle (quintile 3), richer (quintile 4), and richest (quintile 5) [22]. Mass media exposure was created from the three variables: watching television, listening to the radio, and reading a newspaper, and labeled as “yes” if a woman has exposure to either of the three media sources or “no” if a woman has exposure to none of them [23], knowledge about family planning methods was composite variable: if youths know at least one of the following methods: female sterilization, male sterilization, the contraceptive pill, intrauterine contraceptive device (IUD), injectables (Depo Provera), implants, female condom, male condom, diaphragm, contraceptive foam, and contraceptive jelly, lactational amenorrhea method (LAM), standard days method (SDM), country-specific modern methods and respondent-mentioned other modern contraceptive methods (including cervical cap, contraceptive sponge, Periodic abstinence (rhythm, calendar method), withdrawal and country-specific traditional methods were considered as knowledgeable [19].

The countries’ income status was categorized as low-income, lower -middle-income, and upper-middle-income countries based on the World Bank list of economies classification since 2019. The World Bank calculated country income based on Gross National Income (GNI) per capita, which is categorized as low income if GNI is $1025 or less; lower middle income if GNI is $1026–3995; upper middle income if GNI is $3996-12,375, and high income if GNI is $12,375 or more [24]. In this study, the youth is said to have experienced sexual violence if she ever faced sexual violence by her husband/partner, or by anyone other than any husband/partner, and was ever forced to perform unwanted sexual acts.

#### Data management and statistical analysis

This study was performed based on the DHS data obtained from the official DHS measure website. The set of individual recode (IR) data was used to extract the outcome and the independent variables. The DHS data in STATA format was then cleaned, transformed, and append to produce favorable variables for the analysis. The STATA version 16 software was used to generate descriptive and analytic statistics of the appended 33 countries’ data. Before we conducted any statistical analysis, the data were weighted for the sampling probabilities using the weighting factor. The pooled estimate of early sexual initiation among youths in SSA was estimated using a metan STATA command. It was determined using the proportion of early sexual initiation in each SSA country and the standard error which was calculated from the proportion and sample size in each country.

The presence of socioeconomic inequality in some health variables or it is more prominent at some points than others was visualized using a concentration curve [25] and the level of socioeconomic-related inequality in a health variable was measured and compared using a concentration index. [26, 27]. The concentration index has a range of -1 to + 1 and is twice the area between the concentration curve and the line of equity. and the sign indicates the direction of the relationship between early sexual initiation and the distribution of living standards (wealth status). Accordingly, CI = 0 indicated the distribution was proportionate, CI = 1 displayed that the richest person had all of the health variables, whereas CI = − 1 indicated that the poorest person had all of the health variables [28, 29]. However, the outcome variable in the present study is binary (early sexual initiation or not), the bounds of C depend on the mean (µ) of the outcome variable and do not vary between 1 and-1. Thus, the bounds of C vary between µ–1 (lower bound) and 1–µ (upper bound) so the present study used Erreygers normalized concentration index (ECI), which is a modified version of the concentration index [30].

Mathematically, ECI can be defined as:

1 $$\text{ECI}= 4\text{*}{\upmu }\text{*CI}\left(\text{y}\right).$$

Where ECI is Erreygers concentration index, CI(y) is the generalized concentration index and µ is the mean of the health variable, early sexual initiation. Then, the ECI with the standard error (SE) was reported in this study.

c Concentration curves show the cumulative share of the population ranked by living standards, starting with the poorest and ending with the richest (x-axis) to the cumulative percentage of early sexual initiation (y-axis) [29]. The ECI would be a 45^0^-line running from the bottom left-hand corner to the top right-hand corner indicating the absence of Inequality (ECI = 0). Furthermore, the concentration curve lying above and below the equality line (45^0^) indicated that the health variable is disproportionately concentrated between poor (pro-poor or ECI < 0) and rich(pro-rich or ECI > 0), respectively [29, 31]. A concentration curve’s position above or below the line of equality can be determined visually. The ECI and its p-value were calculated to determine the statistical significance of the difference between the concentration curve and the line of perfect equality (45-degree or diagonal line). Decomposition of the ECI was done in order to determine the relative contributions of different factors to the socioeconomic-related inequality of early sexual initiation [29, 31, 32]. For any linear additive regression model of health outcome (y) [29],

2 $$y=\mu +\sum\nolimits _{k}{\beta }_{k}{X}_{k}+\in$$

The concentration index for y, CI, is given as:

3 $$y=\sum\nolimits_k\left(\frac{\beta_k{\overline X}_k}\mu\right)C_k+\frac{{gc}_{\in}}\mu$$

Where “y” is the health outcome variable (in this case socioeconomic related inequality of early sexual initiation), ${X}_{k}$ is a set of the socioeconomic determinants of the health outcome, α is the intercept, ${\beta }_{k}$ is the coefficient of ${X}_{k}$, µ is the mean of y, ${\stackrel{-}{X}}_{k}$ is the mean of ${X}_{k}$, ${C}_{k}$ is the CI for ${X}_{k}$, ${gc}_{\in }$ is the generalized CI for the error term ($\in$), $\frac{{\beta }_{k}{\stackrel{-}{X}}_{k}}{\mu }$ is the elasticity of y with respect to ${\stackrel{-}{X}}_{k}$ [32, 33].

## Result

### Socio-demographic characteristics of respondents

A total of 118,932 female youths were included in this study. Among this, nearly two-thirds (63.45%) of the youths were found in the age group from 20 to 24 years old with a median age of 20(IQR: 4) years, and more than half of the youths (60.20%) were rural residents. About 40.30% had completed secondary school, and 42.17% came from a rich household. Over half of the youth (55.47%) were unmarried, and 65% of the Sub-Saharan African countries included in the study were lower-income (Table 2).

**Table 2:** Socio-demographic characteristics of the female youths in a study of socio-economic inequality of early sexual initiation in Sub-Saharan Africa: based on 2010–2020 DHS

Variable	Category	Weighted frequency	Weighted %
Age	15–19	43,473	36.55
	20–24	75,459	63.45
Residence	Urban	47,335	39.80
	Rural	71,597	60.20
Religion	Christian	76,109	63.99
	Muslim	15,896	13.37
	Other	26,927	22.64
Educational level	No education	27,620	23.22
	Primary	38,492	32.36
	Secondary	47,926	40.30
	Higher	4,894	4.12
Marital status	unmarried	65,970	55.47
	Married	52,962	44.53
Occupation	Not working	57,390	48.25
	Working	61,542	51.75
Ever heard about STI	No	15,249	12.82
	Yes	103,683	87.18
Ever experienced sexual violence	No	113,061	95.06
	Yes	5,871	4.94
Ever chewing Khat	No	115,859	97.42
	Yes	3,073	2.58
Knowledge of FP	Not knowledgeable	5,932	4.99
	Knowledgeable	113,000	95.01
Mass media exposure	No	16,686	14.03
	Yes	102,246	85.97
Wealth index	Poorest	21,499	18.08
	Poorer	23,665	19.90
	Middle	23,613	19.85
	Richer	25,063	21.07
	Richest	25,092	21.10
Regions of SSA	Central	24,845	20.89
	East	40,376	33.95
	West	47,431	39.88
	South	6,280	5.28
Country Level of income	Lower-income	78,236	65.78
	Lower middle income	30,734	25.84
	Upper middle income	9,962	8.38

### The pooled prevalence of early sexual initiation among youths

**Fig. 1:**
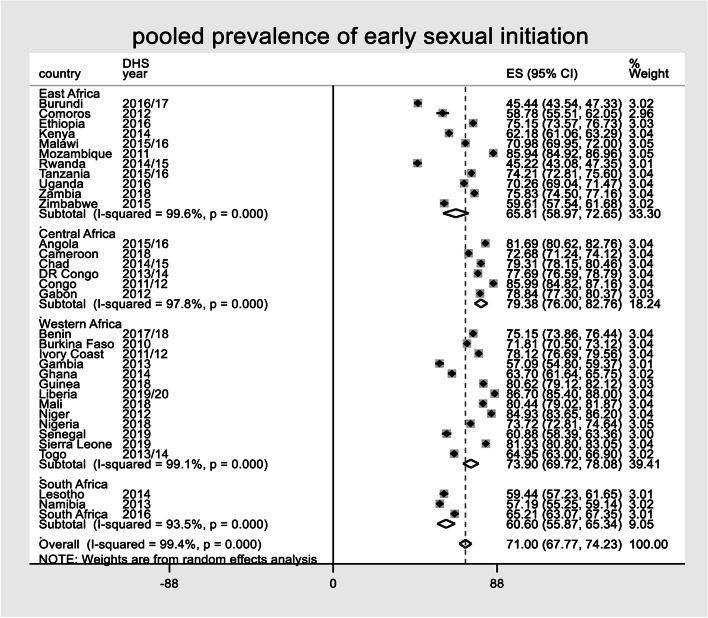
Shows pooled prevalence of early sexual initiation in sub-Sahara Africa by region

The pooled prevalence of early sexual initiation among female youths in Sub-Saharan African countries was 71.00% [95% CI: 67.77 -74.23%] with early sexual initiation being high in Liberia (86.70%), followed by Mozambique and Congo (85.94% and 85.94% respectively); It was lowest in Rwanda (45.22%). Moreover, the pooled prevalence of early sexual initiation among female youths in Eastern African countries was 65.81% (95%CI: 58.97–72.65%), Central African countries 79.38% (95%CI: 76.00-82.76%), Western African countries 73.90 (95%CI: 69.72–78.08%), and 60.60% across Southern African countries (95%CI: 55.87–65.34%) (Fig. 1).

### Socioeconomic inequality of early sexual initiation

**Fig. 2:**
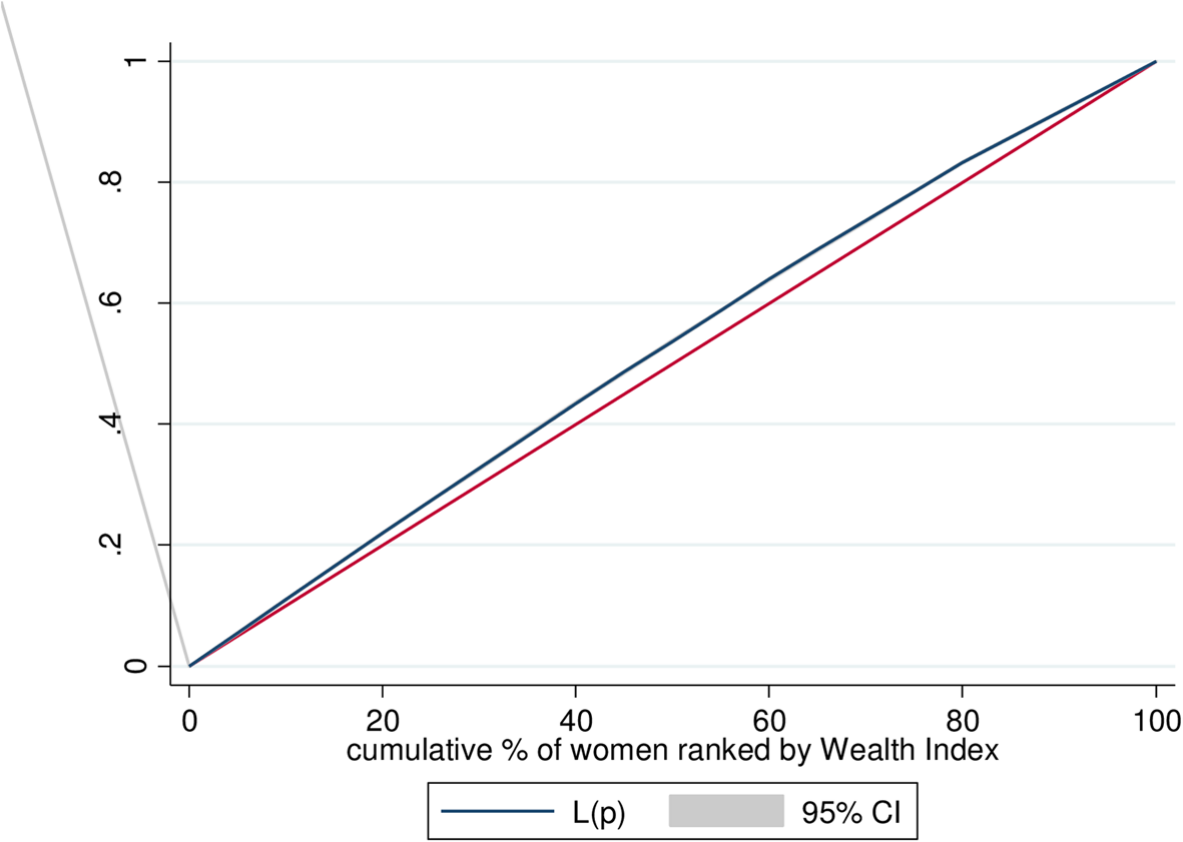
Shows the Concentration curve wealth-related inequality of early sexual initiation in Sub-Saharan Africa

**Fig. 3:**
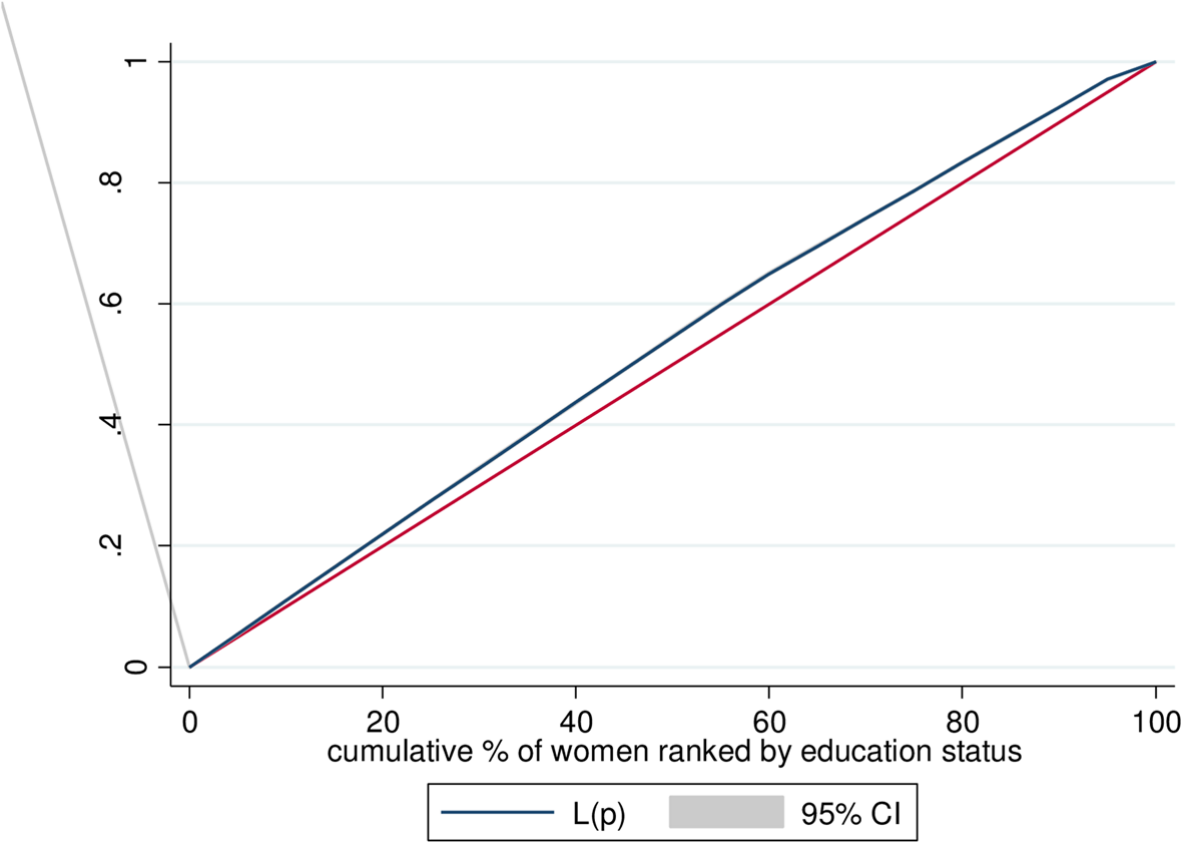
Shows the Concentration curve of educational status-related inequality of early sexual initiation in Sub-Saharan Africa

The weighted Erreygers normalized concentration index (ECI) analysis of wealth-related inequality of early sexual initiation was − 0.157 with Standard error = 0.0046 (*P* value < 0.0001) (Fig. 1). This revealed that early sexual initiation was disproportionately concentrated among the poor (pro-poor). Similarly, the concentration curve showed that the concentration graph of early sexual initiation was above the line of equality which indicated that the distribution of early sexual initiation was concentrated in poor households (-pro-poor distribution) (Fig. 2). Moreover, the weighted Erreygers normalized concentration index (ECI) analysis of educational status-related inequality of early sexual initiation was − 0.205 with Standard error = 0.0043 (*P* value < 0.0001) This revealed that early sexual initiation was disproportionately concentrated among youths with no formal education. Similarly, the concentration curve showed that the concentration graph of early sexual initiation was above the line of equality, which indicated that the distribution of early sexual initiation was concentrated with no formal education (Fig. 3).

### Decomposing the socioeconomic-related inequality in early sexual initiation

After the concentration index and curve were assessed and showed income-related inequality to the early sexual initiation. A decomposition analysis was conducted based on Erreygers normalized concentration index To understand the factors that contribute to socio-economic inequality, the coefficient and its significance level, elasticity, concentration index, and percent contribution were calculated.

Elasticity is the sensitivity of early sexual initiation for each factor. The degree and direction of socioeconomic-related inequality in early sexual initiation that relates to certain explanatory variables are described by the concentration index in each variable. The degree of inequality that the explanatory variables have contributed to can be determined by calculating the absolute contribution by multiplying the elasticity of each factor with its concentration index. Percent contribution means the contribution of each variable to the overall concentration index (Table 3).

**Table 3:** Contributing factors of socio-economic inequality in modern contraceptive utilization in Sub-Sharan Africa

Variables	Category	Coefficient	Elasticity	Concentration index	Absolute contribution	% Contribution
Regions of SSA	Central (ref)	-	-	-	-	-
	East*	-0.0789	-0.1071	-0.0043	0.0005	- 0.318
	West *	-0.0263	-0.0419	-0.0063	0.0003	-0.191
	South *	-0.1138	-0.0240	-0.0007	0.00001	-0.006
Subtotal						**-0.515**
Age	15–19 (ref)	-	-	-	-	-
	20–24*	-0.2685	-0.6813	0.0553	-0.0377	24.01
Religion	Christian	-0.0294	-0.07523	0.0415	-0.0031	1.97
	Muslim	-0.0077	-0.0041	-0.0279	0.0001	0.063
	Others (ref)	-	-	-	-	-
Subtotal						2.033
Residence	Urban (ref)	-	-	-	-	-
	Rural	-0.0094	-0.0226	-0.6453	0.0146	-9.30
Currently working	No (ref)	-	-	-	-	-
	Yes *	-0.0061	-0.0127	-0.1172	0.0015	-0.96
Marital status	Unmarried(ref)	-	-	-	-	-
	Married*	-0.0094	-0.0168	-0.2103	0.0055	-3.50
educational level	no education(ref)	-	-	-	-	-
	Primary	-0.0113	-0.0146	-0.1965	0.0029	-1.85
	Secondary*	-0.1074	-0.1732	0.3327	-0.0576	36.68
	Higher *	-0.2900	-0.0477	0.0971	-0.0046	2.93
Subtotal						**37.76**
Mass media exposure	No (ref)	-	-	-	-	-
	Yes *	0.0374	0.1286	-0.2262	-0.0291	18.53
Experience with sexual violence	No (ref)	-	-	-	-	-
	Yes *	0.0418	0.0083	-0.0125	-0.0001	0.064
Ever heard of STI	No (ref)	-	-	-	-	-
	Yes *	0.0158	0.0553	0.0562	0.0041	-2.61
Ever chewing Khat	No (ref)	-	-	-	-	-
	Yes *	0.1123	0.0116	0.0056	0.0001	-0.064
Wealth index	Poorest (ref)	-	-	-	-	-
	Poorer *	-0.0037	-0.0029	-0.3498	0.0010	-0.64
	Middle *	-0.0292	-0.0231	-0.0333	0.0008	-0.51
	Richer *	-0.0393	-0.0332	0.3096	-0.0103	6.56
	Richest *	-0.0858	-0.0724	0.6659	-0.0452	28.78
Subtotal						**34.19**
Country income level	Lower income (ref)	-	-	-	-	**-**
	Lower middle income*	0.0291	0.0301	-0.0172	-0.0005	0.31
	Upper middle income*	0.0563	0.0188	-0.0058	-0.0001	0.06
Subtotal						**0.37**

Note; *=*p* value < 0.05

In this study, educational status was the major contributing factor to the overall socioeconomic inequality in early sexual initiation (37.76%), followed by the wealth index (34.19%). Moreover, 24.17% of the pro-poor inequalities of early sexual initiation among female youth are explained by age. Nearly 19% of the pro-poor wealth-related inequality for early sexual initiation among female youth was also explained by mass media exposure.

## Discussion

Identifying and reducing avoidable socioeconomic inequalities and other determinants of early sexual initiation is a critical step toward improving youths’ health and well-being. This study aimed to determine the pooled estimate, socioeconomic inequalities of early sexual initiation, and contributing factors among youth in sub-Saharan Africa. According to this study, the pooled prevalence of early sexual initiation among female youths in Sub-Saharan African countries was 71.00% [95% CI: 67.77-74.23%], and early sexual initiation in SSA was disproportionately concentrated among poor households. Evidence based on existing studies [34, 35] supports the findings presented in this study, in relation to how early sexual initiation is inequitable to the disadvantaged and economically poor females who engage in early sexual intercourse in exchange for money and other benefits. This implied that economically disadvantaged female youths engage in early sexual initiation, which increases risks of unintended pregnancy, unsafe abortion, and acquiring HIV/AIDS and other STIs. Therefore, strengthening inter-sectoral collaboration among development sectors is crucial to reduce poverty and engaging in early sexual initiation and promote equity. Contrary to this finding study done in Poland among Polish students, socioeconomic status and living conditions have no influence on the initiation of sex at an earlier age [36].

In this study, educational status was a significant contributor to the overall socioeconomic inequality in early sexual initiation (37.76%). This finding is in line with studies done in East Africa [37], Maynamar [38], Indonesia [39], and Ethiopia [40, 41]. This could be because youth female’s education can result in the corresponding improvement in their level of awareness about reproductive health i.e. about the optimal age for sexual initiation and informed of the consequences of early sexual initiation and related comorbidities which may prevent them from involvement and Education is an essential enabling factor which improves all aspects of youths Reproductive health life [42]. Besides that, education may result in changes in behaviors that reduce possible risks, such as substance use, which may expose them to early sexual initiation [37].

It was found that media exposure was the major and important contributor to the overall socioeconomic inequality in early sexual initiation (18.53%). This finding is in line with studies done in southern India [43], East Africa [37], and Ethiopia [44]. This might be due to respondents who had media exposure may obtain knowledge about the consequence of early initiation of sexual intercourse.

Following educational status, the wealth index also significantly contributed to the overall socioeconomic inequality in early sexual initiation (34.19%). Previous studies had also revealed that wealth is the main determinant factor for early sexual initiation [35, 44–46]. This might be because females from low-income households may participate in earlier sexual relations to obtain money and other benefits, whereas rich people have good health-seeking behavior, awareness of lifestyle determinants, and family traits [37].

This study also revealed that the age between 20 and 24 was another contributor to the socioeconomic inequality in early sexual initiation (24.01%). A previous study also documented that age had strong relationship with early sexual initiation [47]. This might be due to less family supervision as age advanced.

### Strength and limitation

The primary strength of this study was the use of weighted nationally representative data from each Sub-Saharan African country with a large sample, making it representative at both the Sub-Saharan and regional levels. Moreover, the ECI curve and decomposition analysis are appropriate statistical models to show the direction and degree of socioeconomic inequality of early sexual initiation use between the poorest to the richest household. However, because of the cross-sectional nature of the data, the findings are unable to shed light on the temporal relationships between the variables. Therefore, a simple inference could not be made. The information in the survey was also self-reported, so, it was vulnerable to social desirability bias.

## Conclusion and recommendation

The proportion of early sexual initiation among female youths in sub-Saharan Africa was high. Early sexual initiation was disproportionally concentrated in poor households in sub-Saharan Africa (pro-poor concentration). Wealth index, educational status, age, and mass media exposure were the major contributors to pro-poor socioeconomic inequalities of early sexual initiation. Therefore, targeting disadvantaged youths and contributors will help to alleviate these inequalities and improve sexual and reproductive health. This finding will help countries to ensure their disadvantaged populations are not left behind and help policymakers to narrow the disparity of youth sexual health by wealth status. It will also create awareness in the scientific community about the problem and contribute towards formulating locally appropriate strategies to prevent early sexual initiation and it will give information to governmental and non-governmental organizations which work in the area of youth health.

To prevent early sexual initiation among female youths in sub-Saharan Africa, policymakers and other stakeholders should work with other sectors, emphasize, and prioritize achievable variables, such as improving media exposure of the family. For those SSA countries with lower income status needed long-term plans to improve their country’s economy to a higher economic level and to improve the wealth index of individual households. Interventions to reduce early sexual initiation also need balance by supporting marginalized groups such as uneducated female youths.

## Data Availability

The datasets used and/or analyzed for this study are available from the Demographic and Health Surveys (DHS) Program (https://dhsprogram.com/Data/).

## Abbreviations

CI: Concentration Index
DHS: Demographic and Health Survey
ECI: Erreygers Concentration Index
DHS: Demographic and Health Survey
SSA: Sub-Sharan Africa
SDG: Sustainable Development Goal
WHO: World Health Organization
